# Long Live the King!

**Published:** 1902-08

**Authors:** 


A Monthly Review of Medicine and Surgery.

*EDITOR:*

WILLIAM WARREN POTTER, M. D.

All communications, whether of a literary or business nature, books for review and exchanges should be addressed to the editor: 284 FRANKLIN STREET, BUFFALO, N. Y.

VOL. XLII.—LVIII. AUGUST, 1902. NO. 1.

### Long Live the King!

KING EDWARD of England deserves and receives the sympathy and congratulation of the whole civilised world,—sympathy at the grievous illness which laid him low on the eve of his projected coronation, and congratulation upon his recovery with practically complete restoration to health in one short month.

Probably never in the history of the world had such elaborate preparations been made to celebrate the coronation of a monarch as were planned for the ceremony that was to culminate on the 26th of June in crowning at Westminster Abbey Edward VII. and Alexandra as King and Queen of Great Britain and Ireland and Emperor and Empress of India. Probably, too, never had there been two worthier rulers than these to offer themselves for the ecclesiastical and royal ceremony that is by common consent termed a coronation.

There is something in the magnificent splendor of such a scene as was contemplated to be enacted in that classic abbey on that June morning, which appeals to the higher esthetism of a great people. There is in it nothing gross, nothing that savors of oppression, nothing vainglorious, but rather it is a culmination of all that is superb in a splendid twentieth century manhood and womanhood to do honor to the law, to good government, and to wise rulers. For the good king to be stricken down in the midst of such an event, when all the great governments of the earth had assembled their representatives to take part in the ceremonies, was indeed a sore disappointment; not so much to the brave king himself perhaps, as to the loyal, loving people who were proud to do him honor.

We have been led away from the main purpose, predominant in our mind when we began, which was to join our sympathy with that of the great English nation in its disappointment and to express the hope that King Edward VII., may be spared to a ripe old age to rule the destinies of the English people. His training has been such as to prepare him in a special manner for the duties of the crown and he may well be regarded as the foremost monarch of the age.

---

His Majesty is especially fortunate in the choice of his medical staff. Sir Frederick Treves stands at the forefront of the surgical profession, and it is doubtful if there is in the whole world one more competent to make the operation which he did on the King or to conduct the post-operative management. Lord Lister, too, was an excellent adviser and was just such a man as Sir Frederick could lean upon when necessary; for it must be confessed that no surgeon, however competent, would wittingly assume the entire responsibility when such an important life is at stake.

The medical side of the distinguished patient's illness was well served by Sir Thomas Smith, Sir Thomas Barlow and Sir Francis Laking. To these five great men the King owes his life, and a grateful people will not fail to appreciate their skilful management of a case that called forth all the resources of medical and surgical knowledge as well as of physical endurance. His Majesty, himself, has not failed to recognise these facts in the coronation honors he has bestowed. It remains for parliament to perform its part in relation to their pecuniary compensation.

---

The diagnosis of King Edward's disease was given out in the official bulletins as perityphlitis, which in this country we are accustomed to call appendicitis. We are not advised as to the precise nature of the surgical procedure taken, but we infer that its several stages were incision, irrigation, and drainage. If this is correct, the surgical lesion was probably a pericecal abscess resultant from an inflammation in the neighborhood of the vermiform appendix. The depth of the incision must have been great, owing to the thick layer of fatty tissue that covered the King's abdomen and which complicated matters not only as to surgical technic but as to the healing process, which of necessity must be slow in such an operative field. At all events, the result shows that the surgical work was perfect and the post-operative management complete.

---

THE King has bestowed coronation honors upon the following-named medical men: Lord Lister to be one of the first members of the Order of Merit and also a privy councillor; Sir Frederick Treves, knighted in 1901, is advanced to the rank of Baronet; Sir Francis Laking, knighted in 1893, is also advanced to the rank of Baronet; Mr. Victor Horsley, Dr. William Macewen, Dr. Isambard Owen, Dr. Thomas P. Fraser, Dr. J. Halliday Croom, Dr. William Whitla, Mr. Henry Greenway Howse, Mr. Thomas Myles, Dr. William J. Collins, Mr. Alfred Cooper and Dr. A. Conan Doyle, have all received the honor of knighthood. The Order of the Bath (civil) has been conferred upon Sir William Church and that of Commander of the Bath upon Major Ronald Ross, F. R. S., and Dr. B. Arthur Whitelegge. Medical officers of the army and navy also received similar honors.

